# Systemic Immunomodulatory Treatments for Patients With Atopic Dermatitis: A Systematic Review and Network Meta-analysis

**DOI:** 10.1001/jamadermatol.2020.0796

**Published:** 2020-04-22

**Authors:** Aaron M. Drucker, Alexandra G. Ellis, Michal Bohdanowicz, Soudeh Mashayekhi, Zenas Z. N. Yiu, Bram Rochwerg, Sonya Di Giorgio, Bernd W. M. Arents, Tim Burton, Phyllis I. Spuls, Denise Küster, Doreen Siegels, Jochen Schmitt, Carsten Flohr

**Affiliations:** 1 Division of Dermatology, Department of Medicine, University of Toronto, Toronto, Ontario, Canada; 2 Women’s College Research Institute, Department of Medicine, Women’s College Hospital, Toronto, Ontario, Canada; 3 Center for Evidence Synthesis in Health, Brown University, Providence, Rhode Island; 4 Unit for Population-Based Dermatology Research, St John’s Institute of Dermatology, St Thomas’ Hospital, Guy’s and St Thomas’ NHS Foundation Trust, London, United Kingdom; 5 Dermatology Centre, Manchester Academic Health Science Centre, NIHR Manchester Biomedical Research Centre, Salford Royal NHS Foundation Trust, The University of Manchester, Manchester, United Kingdom; 6 Department of Medicine, McMaster University, Hamilton, Ontario, Canada; 7 Departments of Health Research Methods, Evidence and Impact, McMaster University, Hamilton, Ontario, Canada; 8 Libraries & Collections, King’s College London, London, United Kingdom; 9 Dutch Association for People With Atopic Dermatitis, Nijkerk, the Netherlands; 10 Patient Representative (independent), Nottingham, United Kingdom; 11 Department of Dermatology, Amsterdam Public Health, Infection and Immunity, Amsterdam, the Netherlands; 12 Center for Evidence-Based Healthcare, Faculty of Medicine Carl Gustav Carus, Technische Universität Dresden, Dresden, Germany; 13 Unit for Population-Based Dermatology Research, St John’s Institute of Dermatology, St Thomas’ Hospital, London, United Kingdom

## Abstract

**Importance:**

Most clinical trials assessing systemic immunomodulatory treatments for patients with atopic dermatitis are placebo-controlled.

**Objective:**

To compare the effectiveness and safety of systemic immunomodulatory treatments for patients with atopic dermatitis in a systematic review and network meta-analysis.

**Data Sources:**

The Cochrane Central Register of Controlled Trials, MEDLINE, Embase, Latin American and Caribbean Health Science Information database, Global Resource of Eczema Trials database, and clinical trial registries were searched from inception to October 28, 2019.

**Study Selection:**

English-language randomized clinical trials of 8 weeks or more of treatment with systemic immunomodulatory medications for moderate to severe atopic dermatitis were included. Titles, abstracts, and articles were screened in duplicate. Of 10 324 citations, 39 trials were included.

**Data Extraction and Synthesis:**

Data were extracted in duplicate, and the review adhered to Preferred Reporting Items for Systematic Reviews and Meta-analyses for Network Meta-Analyses guidelines. Random-effects bayesian network meta-analyses were performed and certainty of evidence was assessed using Grading of Recommendations Assessment, Development and Evaluation criteria.

**Main Outcomes and Measures:**

Prespecified outcomes were change in signs of disease, symptoms, quality of life, itch, withdrawals, and serious adverse events.

**Results:**

A total of 39 trials with 6360 patients examining 20 medications and placebo were included. Most trials were conducted for adults receiving up to 16 weeks of therapy. Dupilumab, 300 mg every 2 weeks, was associated with improvement in the Eczema Area and Severity Index score vs placebo (mean difference, 11.3-point reduction; 95% credible interval [CrI], 9.7-13.1 [high certainty]). Cyclosporine (standardized mean difference, −1.1; 95% CrI, −1.7 to −0.5 [low certainty]) and dupilumab (standardized mean difference, −0.9; 95% CrI, −1.0 to −0.8 [high certainty]) were similarly effective vs placebo in clearing clinical signs of atopic dermatitis and may be superior to methotrexate (standardized mean difference, −0.6; 95% CrI, −1.1 to 0.0 [low certainty]) and azathioprine (standardized mean difference, −0.4; 95% CrI, −0.8 to −0.1 [low certainty]). Several investigational medications for atopic dermatitis are promising, but data to date are limited to small early-phase trials. Safety analyses were limited by low event rates.

**Conclusions and Relevance:**

Dupilumab and cyclosporine may be more effective for up to 16 weeks of treatment than methotrexate and azathioprine for treating adult patients with atopic dermatitis. More studies directly comparing established and novel treatments beyond 16 weeks are needed and will be incorporated into future updates of this review.

## Key Points

**Question:**

What is the relative effectiveness of systemic treatments for patients with atopic dermatitis?

**Findings:**

This network meta-analysis of 39 randomized clinical trials including 6360 patients found that dupilumab and cyclosporine were similarly effective for adult patients with atopic dermatitis for up to 16 weeks of treatment and were more effective than methotrexate and azathioprine.

**Meaning:**

Cyclosporine and dupilumab may have better short-term effectiveness than methotrexate and azathioprine for patients with atopic dermatitis; this analysis will be updated to add evidence as new medications are approved.

## Teaser

This systematic review and network meta-analysis compares the effectiveness and safety of systemic immunomodulatory treatments for adult patients with atopic dermatitis.

## Introduction

Atopic dermatitis (AD) is a common, chronically relapsing inflammatory skin condition prevalent in 5% to 8% of adults and 11% to 20% of children.^1,2,3^ Approximately one-third of children and half of adults with AD have moderate or severe disease.^1,2^ For those patients, topical treatment and phototherapy may not adequately achieve disease control, requiring systemic therapy.^4^

Systemic immunomodulatory agents used to treat AD include the older medications cyclosporine, methotrexate, azathioprine, and mycophenolate^5^ and the biologic dupilumab.^6^ Numerous biologic and small-molecule medications are being studied in clinical trials.^6^ Understanding the relative effectiveness and safety of different treatments is challenging because most have not been compared head to head. A systematic review of randomized clinical trials (RCTs) published in 2014 did not include these novel therapies or a quantitative synthesis.^7^ The aim of this systematic review and network meta-analysis of RCTs is to assess the relative effectiveness and safety of systemic immunomodulatory therapies for adults and children with moderate-to-severe AD.

## Methods

### Search Strategy and Selection Criteria

We searched the Cochrane Central Register of Controlled Trials, MEDLINE via Ovid (from 1946), Embase via Ovid (from 1974), the Latin American and Caribbean Health Science Information database (from 1982), and the Global Resource of Eczema Trials database. We also performed searches of the following trial registers: the ISRCTN (International Standard Randomised Controlled Trial Number) registry, ClinicalTrials.gov, the Australian New Zealand Clinical Trials Registry, the World Health Organization International Clinical Trials Registry Platform, and the EU Clinical Trials Register. We searched all databases from inception until October 28, 2019. We hand-searched reference lists of relevant publications retrieved as full articles and relevant systematic reviews and literature reviews to identify other eligible studies. The full, updated search strategy can be found at http://eczematherapies.com/research. We searched for supplemental results on trial registries and contacted study authors and pharmaceutical companies to obtain further data. For logistical reasons and because language restriction has not been shown to consistently bias the results of quantitative syntheses, we included only studies published in English.^8^

### Eligibility Criteria

We included studies based on the following eligibility criteria:

#### Population

We included studies of children and adults with moderate-to-severe AD. We applied no age or sex restrictions.

#### Interventions and Comparator

We included studies of systemic (ie, oral, intravenous, or subcutaneous) immunomodulatory therapies for patients with moderate-to-severe AD and any comparator, including placebo.

#### Outcomes

The primary outcomes are (1) change in score on a scale measuring investigator-reported clinical signs, such as the Eczema Area and Severity Index (EASI)^9^; (2) change in score on a scale measuring patient-reported overall symptoms, such as the Patient-Oriented Eczema Measure (POEM)^10^; (3) withdrawal from systemic treatment owing to adverse events; and (4) occurrence of serious adverse events.

The secondary outcomes are (1) change in score on a scale measuring skin-specific health-related quality of life, such as the Dermatology Life Quality Index (DLQI),^11^ and (2) change in score on a scale measuring itch severity.

#### Study Design

We included RCTs of 8 weeks or more of therapy, including 2 doses or more of medication. Although AD is a chronic condition, most trials are of 16 weeks’ duration or less. We included studies with systemic immunomodulatory therapies as monotherapy or in combination with topical therapies. We did not restrict the type of RCT, but for crossover RCTs, we included only outcome data before the crossover.

### Screening and Abstraction Process

We screened titles and abstracts independently in duplicate. Any citation identified by either of the screeners as potentially relevant was advanced to full text review. Two independent investigators (pairs of A.M.D., M.B., S.M., Z.Z.N.Y., P.I.S., D.K., or J.S.) then screened full texts for inclusion; any discrepancies were resolved by discussion between the 2 screeners and, when necessary, with a senior member of the review team (C.F.). All data abstraction was performed in pairs (2 of A.M.D., M.B., S.M., or Z.Z.N.Y.).

We categorized the use of concomitant therapy by whether topical anti-inflammatory treatments (eg, corticosteroids, or calcineurin inhibitors) were permitted. For studies in which patients used topical medications as rescue therapy only and were subsequently excluded from the trial, we categorized the study as not allowing topical therapy. For each outcome, when available, we extracted data for short-term (≤16 weeks) and long-term (>16 weeks) time points. Within each short-term and long-term period, we extracted outcome data at the latest reported end point during active treatment. The duration of active treatment was defined as the time from baseline to the last administered dose plus the interval between doses (eg, if a medication is given every 4 weeks and the last dose is given at 12 weeks, the active-treatment duration is considered 16 weeks). For studies in which safety outcomes were reported only after an off-treatment monitoring period, we assigned those results to the active-treatment time frame so that our analysis reflected the treatment exposure (eg, for 16 weeks of active treatment with safety follow-up reported at 24 weeks, we considered those safety data to pertain to 16 weeks of treatment). For studies in which the analytic population for an outcome differed from the baseline population, and baseline values for the outcome measure were provided only for the baseline population, we used that baseline value.

For effectiveness outcomes, the relevant data included the mean change from baseline and a measure of variance. If data were not reported as change from baseline, we used the mean baseline and follow-up values. When mean percentage change from baseline was reported, we converted the data to mean change from baseline if baseline values were provided, assuming equal variances at baseline and follow-up and a correlation of 0.5 between baseline and follow-up.^12^ For withdrawals and serious adverse events, we extracted the number of individuals experiencing the event and the number included in the analysis. If results were available only in figures with exact values not given, we used Engauge Digitizer software, version 10.11 to estimate the values.^13^ Two reviewers (A.M.D. and M.B.) derived estimates independently, and we used the mean of their estimates.

### Statistical Analysis

We performed network meta-analysis for each outcome using a random-effects model within a bayesian framework using the *gemtc* package in R, version 3.6 (R Foundation for Statistical Computing).^14^ For continuous outcomes, the model corresponds to a generalized linear model with an identity link.^15^ For binary outcomes, the model corresponds to a generalized linear model with a logit link.^15^ We included random effects on the treatment parameters, which allows each study to have a different but related treatment effect. The between-study variance (heterogeneity) was assumed to be constant for every treatment comparison. We used noninformative prior distributions for effectiveness model parameters given current uncertainty of the relative effectiveness of the treatments.^4^ Because of the sparseness of data for withdrawals and serious adverse events, we conducted analyses using a more informative log-normal prior for the heterogeneity parameter.^16^ Specifically, we assumed a reasonable bound would capture the treatment effects because, in the context of pharmaceutical interventions, it is very unlikely that an odds ratio will be greater than 30 or less than 1/30.^17^ Hence, we used a normal prior on the log odds ratios such that the 95% coverage includes log (1/30) to log (30). We assessed the convergence of 4 chains using the Gelman-Rubin statistic and by visual inspection of trace plots. We planned to assess coherence (also called *consistency*) by comparing the direct and indirect evidence using a node-splitting approach.^18^ However, the geometry of our networks, particularly the paucity of head-to-head trials, precluded planned evaluations of consistency at this stage. Once sufficient evidence is available to evaluate consistency, we will do so in future updates of our network meta-analysis.

We planned to pool studies of 8 to 16 weeks of treatment separately from studies of more than 16 weeks of treatment and studies of children separately from studies of adults. A paucity of long-term and pediatric trials limited analyses to short-term trials for adults. For medications studied at different doses, we treated each dosing regimen as its own network node so that suboptimal doses in early trials did not bias the effect estimates for doses ultimately used in practice. Within each outcome domain, we analyzed each scale separately when there were sufficient data. The published estimate of the minimal clinically important difference is 6.6 for the EASI score,^9^ 3.4 for the POEM score,^10^ 3.3 for the DLQI score,^11^ and 2.6 for the Peak Pruritus Numeric Rating Scale score ^19,20,21^; we used these minimal clinically important differences as indicators of clinical significance and to guide Grading of Recommendations Assessment, Development and Evaluation (GRADE) imprecision assessments.

In separate analyses, we combined all scales within each outcome domain using the standardized mean difference (SMD). The EASI, DLQI, POEM, and Peak Pruritus Numeric Rating Scale are in the AD core outcome set, so we prioritized the data from these outcome measures.^10,22^ For these analyses, we interpreted summary effect estimates less than 0.2 as small, 0.2 to 0.8 as moderate, and more than 0.8 as large.^23^

We generated network plots for each analysis. Summary results are presented as the mean difference, SMD, or odds ratio with 95% credible intervals (CrIs). Treatment rankings are summarized by the surface under the cumulative ranking that expresses the percentage (0%-100%) of effectiveness or safety each treatment has compared with an ideal treatment ranked always first without uncertainty.^24^

### Subgroup and Sensitivity Analyses

We conducted post hoc subgroup analyses separating trials that allowed vs did not allow concomitant topical anti-inflammatory therapy. We performed a post hoc secondary analysis including only treatments currently in widespread use. These treatments include the labeled adult dosage of dupilumab (600 mg for 1 dose then 300 mg every 2 weeks), lower-dose cyclosporine (≤3 mg/kg/d and 150 mg/d), higher-dose cyclosporine (>3 mg/kg/d and ≤5 mg/kg/d and 300 mg/d), and various similar azathioprine and methotrexate dosage regimens.

We conducted planned sensitivity analyses including only trials found to be at low risk of bias (no items scored as unclear or high risk of bias). We also conducted sensitivity analyses including only studies reporting mean change from baseline, excluding those reporting mean change at follow-up or mean percentage change. In another analysis, for the conversion of mean percentage change to mean change, we relaxed the assumption of equal variances at baseline and follow-up and calculated the follow-up SD as the product of the baseline SD and the ratio of the mean SD at follow-up to baseline observed among studies with reported SDs.

### Risk of Bias and Certainty of Evidence

We assessed the risk of bias in individual studies using the Cochrane Risk of Bias tool.^25^ Assessments were done in duplicate (A.M.D. and either D.K. or D.S. supervised by J.S.); discrepancies were resolved by discussion between assessors. To empirically assess for publication bias, we compared the results of our trial registry searches with the results from published studies.

We assessed the overall quality of evidence for each outcome and comparison using GRADE criteria for the network meta-analysis estimates.^26,27^ Imprecision for each comparison was assessed at the network meta-analysis level only. The certainty for indirect estimates was inferred from examination of the dominant first-order loops and is the lowest of the direct estimates contributing. We further lowered our quality assessment for intransitivity if there were important differences between studies forming the dominant first-order loop.

### Protocol

The research plan was developed by dermatologists, patients with AD, methodologists, and an information specialist. We registered a protocol in PROSPERO (CRD42018088112), and it was published in full.^28^ After the publication of the protocol but prior to data analysis, we decided to exclude studies with treatments given only as a single dose, with active treatment less than 8 weeks, with a run-in period that included use of a systemic immunomodulatory agent, or when results were available only in a clinical trial registry for studies terminated early of medications not approved. These changes were made to improve homogeneity and decrease intransitivity.

### Living Systematic Review

As this area of research is rapidly evolving with new trials being added to the literature regularly, we will continuously update our findings as a living systematic review and network meta-analysis. We will update our search and analysis every 4 months, which is considered feasible for rapidly evolving fields.^29^

The living systematic review will be updated at a website (http://eczematherapies.com/research) hosted by one of our (A.M.D.) institutions, developed with funding from peer-reviewed grants and independent from industry. We will publish major updates in peer-reviewed journals.

## Results

### Included Studies

**Figure 1.**
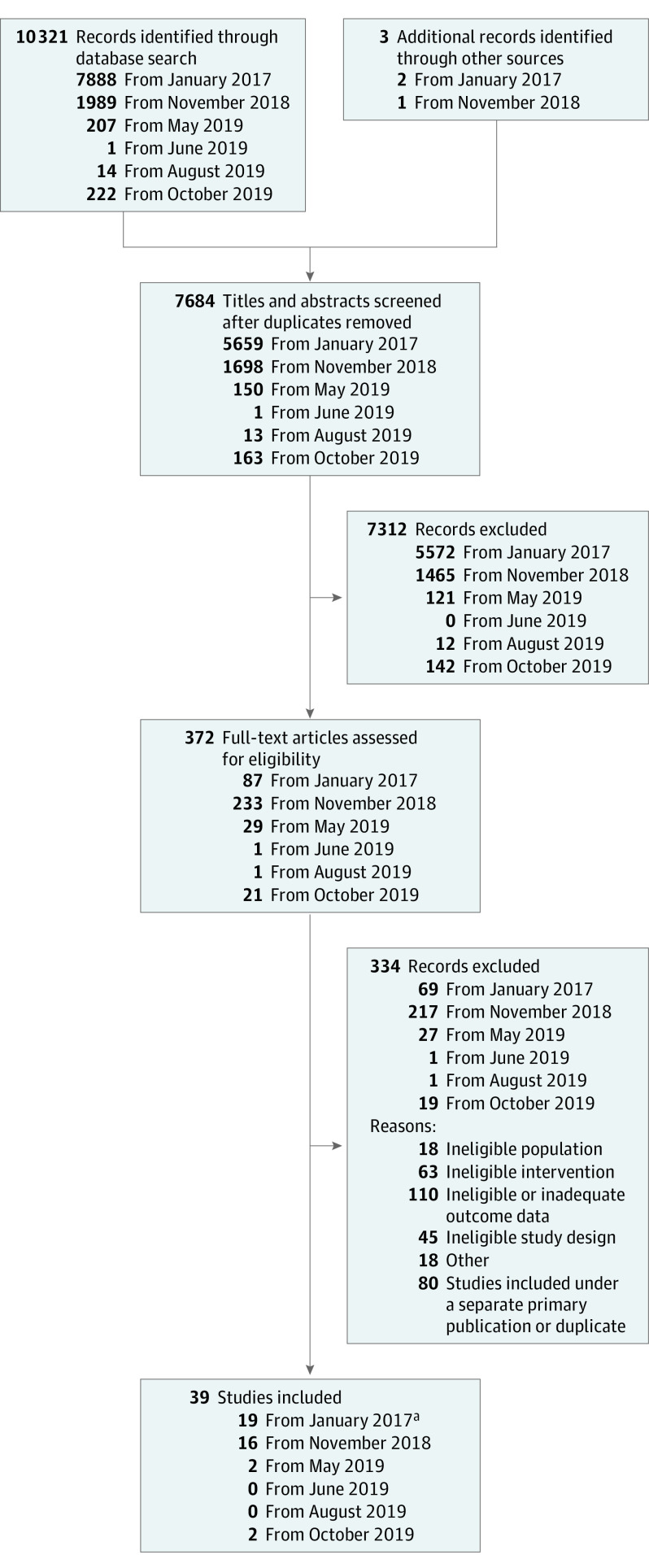
Study Selection Process ^a^Dupilumab SOLO (Study of Dupilumab [REGN668/SAR231893] Monotherapy Administered to Adult Patients With Moderate-to-Severe Atopic Dermatitis) 1 and SOLO 2 studies were published in a single article.

Our searches yielded 10 321 titles, abstracts, and trial registry entries, as well as 3 studies identified from other sources; we ultimately included 39 trials with 6360 patients (Figure 1). The included studies evaluated 20 different systemic immunomodulatory therapies and most comparisons were with placebo. Study characteristics and extracted outcomes data are found at http://eczematherapies.com/research. Mean sample size per group was 60 (range, 4-319), the mean proportion of females per trial group was 45%, and the mean or median age in trial groups ranged between 6 and 44 years. Most trials (n = 29) were sponsored by industry. Two trials were reported together in a single manuscript,^30^ 1 study was reported in 2 abstracts only,^31,32^ 1 study was reported in an abstract and trial registry,^33,34^ and 1 study was reported exclusively in a trial registry.^35^ We obtained unpublished data in personal communications for 3 trials.^36,37,38^

Very few studies (n = 6) included outcomes beyond 16 weeks, and network meta-analyses were therefore limited to short-term outcomes. Sixteen studies had at least 1 element at high risk of bias (eTable 1 in the Supplement). There were generally fewer elements at high risk of bias and unclear risk of bias in newer studies compared with older studies. Most recent studies, compared with older studies, had a low risk of bias associated with blinding, but incomplete outcome data were a potential source of bias even among some recently published studies. Each included study contained data on at least 1 outcome of interest (effectiveness or safety). During full-text screening we excluded 103 citations, mainly trial registry entries and abstracts, owing to no outcomes data or insufficient outcomes data being available. Many included studies did not have data on all outcomes, including some outcomes that were prespecified, indicating possible outcome reporting bias.

**Figure 2.**
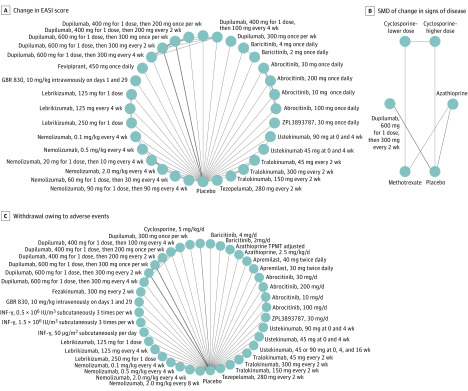
Network Graphs of Studies Included in the Analysis of Atopic Dermatitis Treatment Between 8 and 16 Weeks A, Change in Eczema Area and Severity Index (EASI) score. B, The standardized mean difference (SMD) of change in signs of the disease among medications currently in use. C, Withdrawal owing to adverse events. The width of each line connecting 2 treatments (nodes) is proportional to the number of head-to-head trials for that comparison. INF indicates interferon; and TPMT, thiopurine methyltransferase.

Our network graphs generally show placebo connected to multiple nodes, connections between nodes of different doses of the same medication, and only a few direct connections between different active medications (Figure 2). Subgroup and secondary analyses for effectiveness outcomes according to use of topical anti-inflammatory treatment, risk of bias, and outcome presentation generally decreased the number of trials included in the analysis, resulting in fewer included medications and decreased precision without substantially altering effect estimates. Network graphs and league, Surface Under the Cumulative Ranking, and GRADE tables for all analyses, including sensitivity and subgroup analyses, are available at http://eczematherapies.com/research.

### Change in Clinical Signs

**Figure 3.**
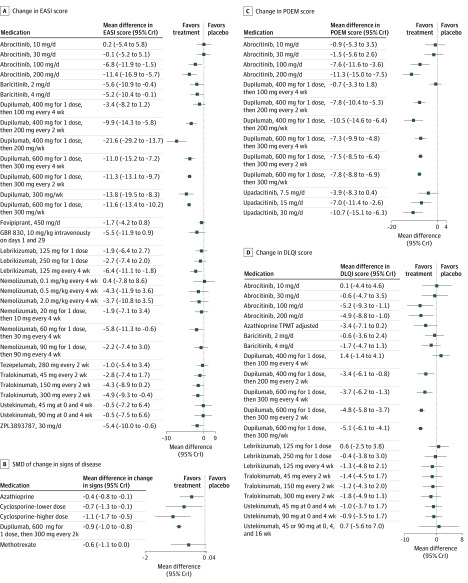
Forest Plots of Network Meta-analysis Results for Atopic Dermatitis Treatment Between 8 and 16 Weeks A, Change in Eczema Area and Severity Index (EASI) score. B, The standardized mean difference (SMD) of change in signs of the disease among medications currently in use. C, Change in Patient-Oriented Eczema Measure (POEM) score. D, Change in Dermatology Life Quality Index (DLQI) score. Results for fevripitant are available only in clinical trial registries. The SEs given for the change in EASI score are very small and may be in error. If in fact these SEs are erroneous, they may have changed the precision of the effect estimates for comparisons with fevripirant. Each systemic medication is compared with placebo. Negative values represent improvement in the disease state.

Figure 2A and Figure 3A show the results for the mean change in EASI score. Dupilumab 300 mg every 2 weeks (the approved dosage for adults) was superior to placebo (mean difference, 11.3-point reduction; 95% CrI, 9.7-13.1 [GRADE assessment: high certainty]). Several investigational medications demonstrated reduction in EASI score compared with placebo, including baricitinib, 2 mg daily (mean difference, 5.6-point reduction; 95% CrI, 0.4-10.9 [GRADE assessment: moderate certainty]) and 4 mg daily (mean difference, 5.2-point reduction; 95% CrI, 0.1-10.4 [GRADE assessment: moderate certainty]), and tralokinumab, 150 mg every 2 weeks (mean difference, 4.3-point reduction; 95% CrI, –0.2 to 8.9 [GRADE assessment: moderate certainty]) and 300 mg every 2 weeks (mean difference, 4.9-point reduction; 95% CrI, 0.4-9.3 [GRADE assessment: moderate certainty]).

Figure 2B and Figure 3B show the results for the analysis of the SMD in change in clinical signs limited to medications currently in widespread clinical use. Azathioprine, lower-dose cyclosporine, higher-dose cyclosporine, methotrexate, and dupilumab had moderate or large benefits relative to placebo. Higher-dose cyclosporine (SMD, −1.1; 95% CrI, −1.7 to −0.5 [low certainty]) and dupilumab (SMD, −0.9; 95% CrI, −1.0 to −0.8 [high certainty]) were similarly effective vs placebo in clearing clinical signs of AD and may be superior to methotrexate (SMD, −0.6; 95% CrI, −1.1 to 0.0 [low certainty]) and azathioprine (SMD, −0.4; 95% CrI, −0.8 to −0.1 [low certainty]). Higher-dose cyclosporine may be associated with improvement in clinical signs compared with azathioprine (SMD, −0.6; 95% CrI, −1.2 to 0.0 [low certainty]) and methotrexate (SMD, −0.5; 95% CrI, −1.1 to 0.0 [low certainty]), with similar improvement to dupilumab (SMD, −0.2; 95% CrI, −0.8 to 0.4 [low certainty]).

### Change in Patient-Reported Outcomes

Dupilumab, 300 mg every 2 weeks (mean difference, –7.5; 95% CrI, –8.5 to –6.4 [high certainty]), and investigational drugs abrocitinib, 100 mg daily (mean difference, –7.6; 95% CrI, –11.6 to –3.6 [low certainty]) and 200 mg daily (mean difference, –11.3; 95% CrI, –15.0 to –7.5 [low certainty]), and upadacitinib, 15 mg daily (mean difference, –7.0; 95% CrI, –11.4 to –2.6 [low certainty]) and 30 mg daily (mean difference, –10.7; 95% CrI, –15.1 to –6.3 [low certainty]) were associated with clinically relevant improvements in the POEM score compared with placebo (Figure 3C; eFigure 1 in the Supplement). Dupilumab, 300 mg every 2 weeks (mean difference, –4.8; 95% CrI, –5.8 to –3.7 [high certainty]), and abrocitinib, 100 mg daily (mean difference, –5.2; 95% CrI, –9.3 to –1.1 [low certainty]) and 200 mg daily (mean difference, –4.9; 95% CrI, –8.8 to –1.0 [low certainty]), were associated with clinically important differences in the DLQI score compared with placebo (Figure 3D; eFigure 2 in the Supplement). Azathioprine dosed according to thiopurine methyltransferase levels was associated with clinically meaningful improvement in the DLQI score compared with placebo, but this improvement was based on low-certainty evidence owing to imprecision (mean difference, −3.4; 95% CrI, −7.1 to 0.2). Comparisons between cyclosporine, dupilumab, methotrexate, and azathioprine in improvement in quality of life on the SMD scale were imprecise (eFigure 3 and eTable 2 in the Supplement).

In the analysis of SMDs in change in itch scales, cyclosporine, 5 mg/kg daily (SMD, −0.8; 95% CrI, −1.7 to 0.1 [very low certainty]), and dupilumab, 300 mg every 2 weeks (SMD, −0.8; 95% CrI, −1.0 to −0.7 [high certainty]), were associated with improvements in itch relative to placebo. Comparisons between cyclosporine, dupilumab, methotrexate, and azathioprine on the SMD scale for itch were imprecise (eFigure 4 and eTable 3 in the Supplement).

### Safety

Given low adverse event rates, robust, interpretable relative safety estimates, particularly among medications currently in use, are not possible. Many of the studies reported 0 events for 1 or more treatments, which generates results that cannot be estimated or results with high uncertainty, even in our analyses with more informative priors.

## Discussion

This network meta-analysis is based on 39 RCTs including 6360 patients taking 20 systemic AD medications. In analyses of outcomes in adult patients receiving between 8 and 16 weeks of treatment, dupilumab was efficacious based on high-certainty evidence with regards to improving clinical signs, including clinically important differences in EASI scores. Dupilumab and the investigational Janus kinase inhibitors upadacitinib and abrocitinib provided clinically meaningful improvement in POEM scores and dupilumab and abrocitinib were associated with clinically meaningful improvements in the DLQI score compared with placebo.

Our analyses using the SMD scale permitted comparisons of dupilumab with older systemic AD medications, for which no head-to-head trials exist, to our knowledge. Dupilumab and higher-dose cyclosporine appear to have better effectiveness during the first 4 months of therapy in improving clinical signs, itch, and quality of life relative to methotrexate and azathioprine. These analyses are limited by pooling outcome measures such as peak itch and mean itch, which measure the same domain but in different ways, and their inclusion of trials only up to 16 weeks, which may favor medications with more rapid onset of action. Despite these concerns and low certainty according to GRADE, our stratification of the currently available treatments should be useful to stakeholders including patients, clinicians, guideline developers, and health technology assessors.

### Limitations

This study has some limitations. There is some heterogeneity in the design of the included trials. In particular, use of background therapy (topical anti-inflammatory medications) differed between studies, which could affect the transitivity assumption. We accounted for some of this heterogeneity through sensitivity analyses and did not observe substantial changes in our findings. For example, the effect estimates for difference in change in EASI score between the approved dose of dupilumab vs placebo were similar in the analyses including trials with vs without background topical anti-inflammatory therapy. All studies included patients with moderate-to-severe AD who were eligible for systemic immunomodulatory therapy, which is reassuring regarding the transitivity assumption. However, studies used different definitions of severity, and while most studies included patients with both moderate and severe AD, some include patients with only moderate or only severe AD. Most comparisons were informed by only a single RCT and usually with a small number of patients, which led to imprecision. Although language restriction can lead to important data being omitted from systematic reviews, our pragmatic decision to limit inclusion to English-language publications did not meaningfully affect the results of our network meta-analysis. We excluded only 1 study on the basis of language, a trial comparing cyclosporine with transfer factor, a treatment not used in clinical practice. Our networks are sparse and most trials were placebo-controlled, which limited our power to estimate heterogeneity and statistical incoherence. Future primary studies with head-to-head comparisons are necessary to improve the evidence base. Our safety analyses were uninformative and future updates including studies with larger sample sizes and longer duration may improve our ability to detect differences in safety and tolerability. There is a Cochrane review under way with similar objectives and methods to ours. Based on their published protocol, their review differs from ours in that it does not include plans for regular updating.^39^

## Conclusions

Cyclosporine and dupilumab may have better short-term effectiveness than methotrexate and azathioprine for treatment of AD in adults. In the absence of well-powered head-to-head trials comparing all possible combinations of active treatments, our study provides the best available comparative effectiveness estimates to inform treatment decisions, guidelines, and health technology assessments. Ongoing and planned RCTs will give more precision to our effect estimates and provide estimates for children and longer-term outcomes.

## Notes

Supplement. **eTable 1.** Risk of Bias in Trials **eTable 2.** League Table of Arms Included in the Network Meta-analysis of Change in QoL on the Standardized Mean Difference Scale up to 16 Weeks of Treatment Among Medications Currently in Use **eTable 3.** League Table of Arms Included in the Network Meta-analysis of Change in Itch on the Standardized Mean Difference Scale up to 16 Weeks of Treatment Among Medications Currently in Use **eFigure 1.** Network Plot of Arms Included in the Network Meta-analysis of Change in POEM Score up to 16 Weeks of Treatment – Main Analysis **eFigure 2.** Network Plot of Arms Included in the Network Meta-analysis of Change in DLQI Score up to 16 Weeks of Treatment – Main Analysis **eFigure 3.** Network Plot of Arms Included in the Network Meta-analysis of Change in QoL on the Standardized Mean Difference Scale up to 16 Weeks of Treatment – Among Medications Currently in Use **eFigure 4.** Network Plot of Arms Included in the Network Meta-analysis of Change in Itch on the Standardized Mean Difference Scale up to 16 Weeks of Treatment – Among Medications Currently in Use **eReferences.**
