# Supplementary material for: Systemic Immunomodulatory Treatments for Patients With Atopic Dermatitis: A Systematic Review and Network Meta-analysis
Source: JAMA Dermatol. 2020 Apr 22;156(6):1–10. doi: 10.1001/jamadermatol.2020.0796 (PMC7177646; doi:10.1001/jamadermatol.2020.0796)
Supplement: Supplement. — eTable 1. Risk of Bias in Trials eTable 2. League Table of Arms Included in the Network Meta-analysis of Change in QoL on the Standardized Mean Difference Scale up to 16 Weeks of Treatment Among Medications Currently in Use eTable 3. League Table of Arms Included in the Network Meta-analysis of Change in Itch on the Standardized Mean Difference Scale up to 16 Weeks of Treatment Among Medications Currently in Use eFigure 1. Network Plot of Arms Included in the Network Meta-analysis of Change in POEM Score up to 16 Weeks of Treatment – Main Analysis eFigure 2. Network Plot of Arms Included in the Network Meta-analysis of Change in DLQI Score up to 16 Weeks of Treatment – Main Analysis eFigure 3. Network Plot of Arms Included in the Network Meta-analysis of Change in QoL on the Standardized Mean Difference Scale up to 16 Weeks of Treatment – Among Medications Currently in Use eFigure 4. Network Plot of Arms Included in the Network Meta-analysis of Change in Itch on the Standardized Mean Difference Scale up to 16 Weeks of Treatment – Among Medications Currently in Use eReferences. [file jamadermatol-156-659-s001.pdf]

Supplementary Online Content

Drucker AM, Ellis AG, Bohdanowicz M, et al. Systemic immunomodulatory treatments for patients with atopic dermatitis: a systematic review and network meta-analysis. *JAMA Dermatol.* Published online April 22, 2020. doi:10.1001/jamadermatol.2020.0796

eTable 1. Risk of Bias in Trials
eTable 2. League Table of Arms Included in the Network Meta-analysis of Change in QoL on the Standardized Mean Difference Scale up to 16 Weeks of Treatment Among Medications Currently in Use
eTable 3. League Table of Arms Included in the Network Meta-analysis of Change in Itch on the Standardized Mean Difference Scale up to 16 Weeks of Treatment Among Medications Currently in Use
eFigure 1. Network Plot of Arms Included in the Network Meta-analysis of Change in POEM Score up to 16 Weeks of Treatment – Main Analysis
eFigure 2. Network Plot of Arms Included in the Network Meta-analysis of Change in DLQI Score up to 16 Weeks of Treatment – Main Analysis
eFigure 3. Network Plot of Arms Included in the Network Meta-analysis of Change in QoL on the Standardized Mean Difference Scale up to 16 Weeks of Treatment – Among Medications Currently in Use
eFigure 4. Network Plot of Arms Included in the Network Meta-analysis of Change in Itch on the Standardized Mean Difference Scale up to 16 Weeks of Treatment – Among Medications Currently in Use
eReferences.

This supplementary material has been provided by the authors to give readers additional information about their work.

© 2020 Drucker AM et al. *JAMA Dermatology*.

eTable 1. Risk of Bias in Trials

Reference	Random sequence generation	Allocation concealment	Blinding of participants and personnel	Blinding of outcome assessment	Incomplete outcome data	Selective reporting	Other bias
Sowden UK 1991[1]	Unclear	Unclear	Low	Low	High	Unclear	Low
Hanifin USA 1993[2]	Low	Unclear	Low	Unclear	High	Unclear	Low
Munro UK 1994[3]	Unclear	Unclear	Low	Unclear	High	Unclear	Unclear
Czech Germany 2000[4]	Low	Unclear	Unclear	Unclear	Low	Unclear	Unclear
Jang Korea 2000[5]	Unclear	Unclear	Unclear	Unclear	Unclear	Unclear	Unclear
Granlund Finland 2001[6]	Unclear	Unclear	High	High	Unclear	Unclear	Low
Berth-Jones UK 2002[7]	Low	Unclear	Low	Unclear	High	Unclear	High
Pacor Italy 2004[8]	Unclear	Unclear	Low	Low	Low	Unclear	Unclear
Bemanian Iran 2005[9]	Unclear	Unclear	High	High	High	Unclear	Unclear
Meggit UK 2006[10]	Low	Unclear	Low	Unclear	Unclear	Unclear	Low
Jee Korea 2011[11]	Unclear	Unclear	Unclear	Unclear	High	Unclear	High
Schram Holland 2011[12]	Low	Low	High	Unclear	Low	Low	Low
El-Khalawany Egypt 2013[13]	Low	Unclear	High	High	Low	Low	Low
Iyengar USA 2013[14]	Unclear	Unclear	Unclear	Unclear	Low	High	Low

© 2020 Drucker AM et al. *JAMA Dermatology*.

Reference	Random sequence generation	Allocation concealment	Blinding of participants and personnel	Blinding of outcome assessment	Incomplete outcome data	Selective reporting	Other bias
Beck USA 2014[15,16]	Low	Low	Low	Low	Low	Low	Low
Khattri USA 2017[17]	Low	Low	Low	Low	Low	Low	Low
Simpson USA 2016 SOLO1[18]	Low	Low	Low	Low	Low	Low	Low
Simpson USA 2016 SOLO2[18]	Low	Low	Low	Low	Low	Low	Low
Thaci Germany 2015[19,20]	Low	Low	Low	Low	Low	Low	Low
Blauvelt USA 2017[21]	Low	Low	Low	Low	Low	Low	Low
Goujon France 2018[22]	Low	Low	High	Low	Unclear	Low	Low
Ruzicka Germany 2017[23]	Low	Low	Low	Low	High	Low	Low
Saeki Japan 2017[24]	Low	Low	Low	Low	Low	Low	Low
Upadacitinib abstracts 2019[25,26]	Unclear	Unclear	Low	Low	Low	Unclear	Low
de Bruin-Weller Netherlands 2018[27]	Low	Low	Low	Low	Low	Low	Low
Guttman-Yassky USA 2018 Fezakinumab[28]	Unclear	Unclear	Low	Low	Low	Low	Low
Guttman-Yassky USA 2018 Baricitinib[29]	Low	Low	Low	Low	Low	Low	Low
Simpson USA 2018 Lebrikizumab[30]	Low	Unclear	Low	Low	Low	Low	Low

© 2020 Drucker AM et al. *JAMA Dermatology*.

Reference	Random sequence generation	Allocation concealment	Blinding of participants and personnel	Blinding of outcome assessment	Incomplete outcome data	Selective reporting	Other bias
Wollenberg USA 2018[31]	Low	Low	Low	Low	Low	Low	Low
Blauvelt USA 2018[32]	Low	Low	Low	Low	Unclear	Low	Low
Guttman-Yassky USA 2019 Dupilumab[33]	Low	Low	Low	Low	Unclear	Low	Low
Guttman-Yassky USA 2019 GBR 830[34]	Low	Low	Low	Low	Low	High	Low
Simpson USA 2018 Apremilast[35]	Low	Low	Low	Low	Low	Unclear	Low
Simpson USA 2019 Tezepelumab[36]	Low	Low	Low	Low	High	Low	Low
Werfel Germany 2018[37]	Low	Low	Low	Low	High	Low	Low
NCT01785602; Fevipiprant (QAW039)[38]	Unclear	Unclear	Low	Low	Unclear	Low	Low
Gooderham Canada 2019[39,40]	Low	Low	Low	Low	High	Unclear	Low
Paller USA 2019 Dupilumab[41,42]	Unclear	Unclear	Low	Low	Low	Low	Low
Silverberg USA 2019 Dupilumab[43,44]	Low	Low	Low	Low	Unclear	Low	Low

Reasons for risk of bias assessment can be found at eczematherapies.com/research.

© 2020 Drucker AM et al. *JAMA Dermatology*.

eFigure 1. Network Plot of Arms Included in the Network Meta-analysis of Change in POEM Score up to 16 Weeks of Treatment – Main Analysis

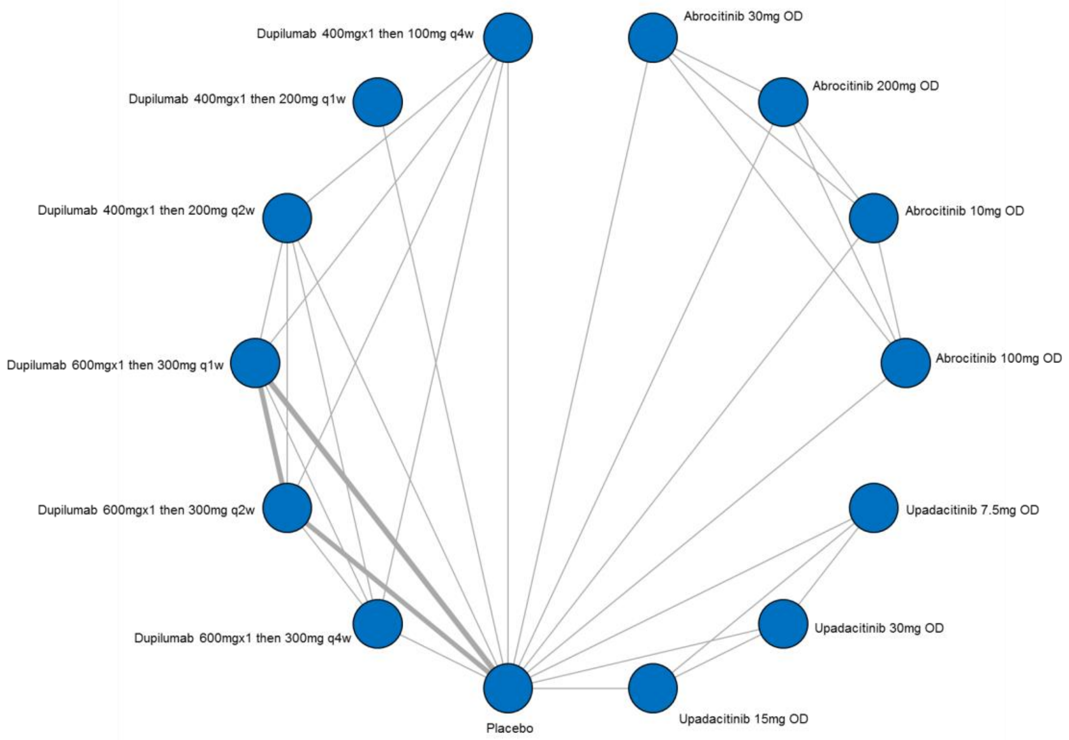

© 2020 Drucker AM et al. *JAMA Dermatology*.

eFigure 2. Network Plot of Arms Included in the Network Meta-analysis of Change in DLQI Score up to 16 Weeks of Treatment – Main Analysis

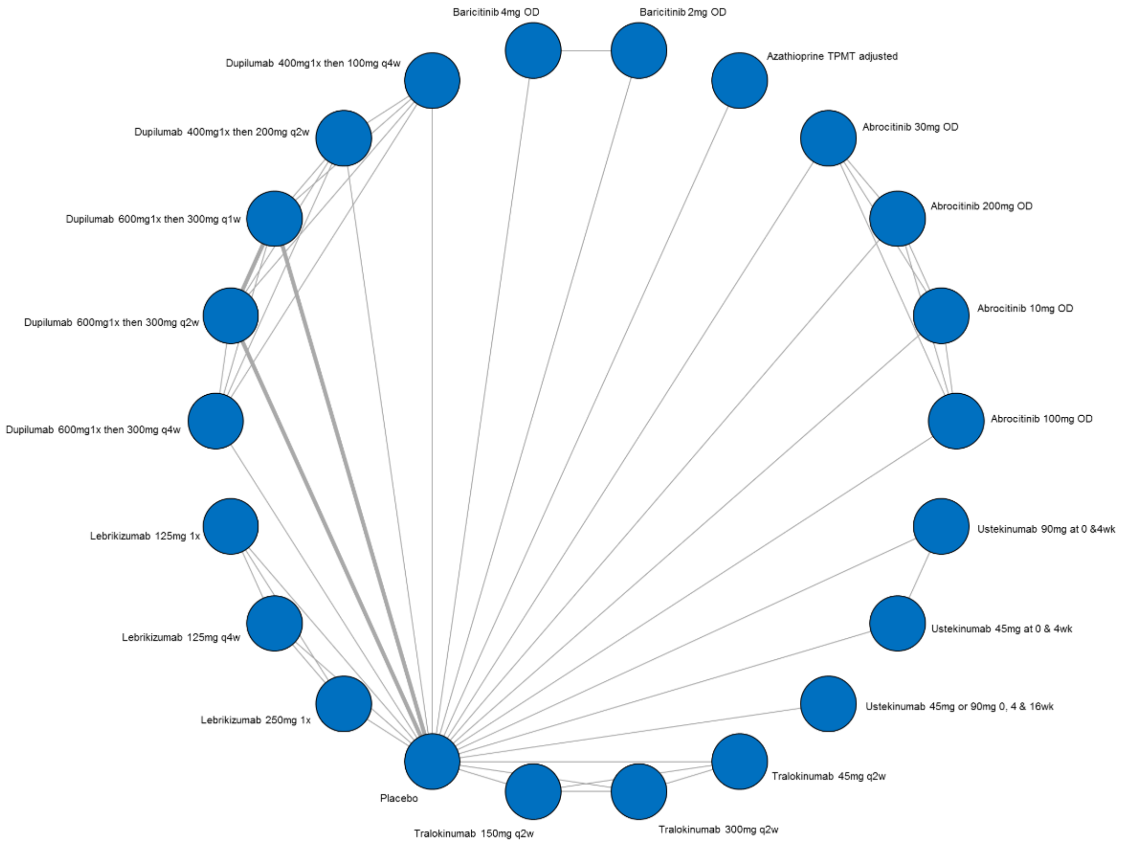

© 2020 Drucker AM et al. *JAMA Dermatology*.

eFigure 3. Network Plot of Arms Included in the Network Meta-analysis of Change in QoL on the Standardized Mean Difference Scale up to 16 Weeks of Treatment – Among Medications Currently in Use

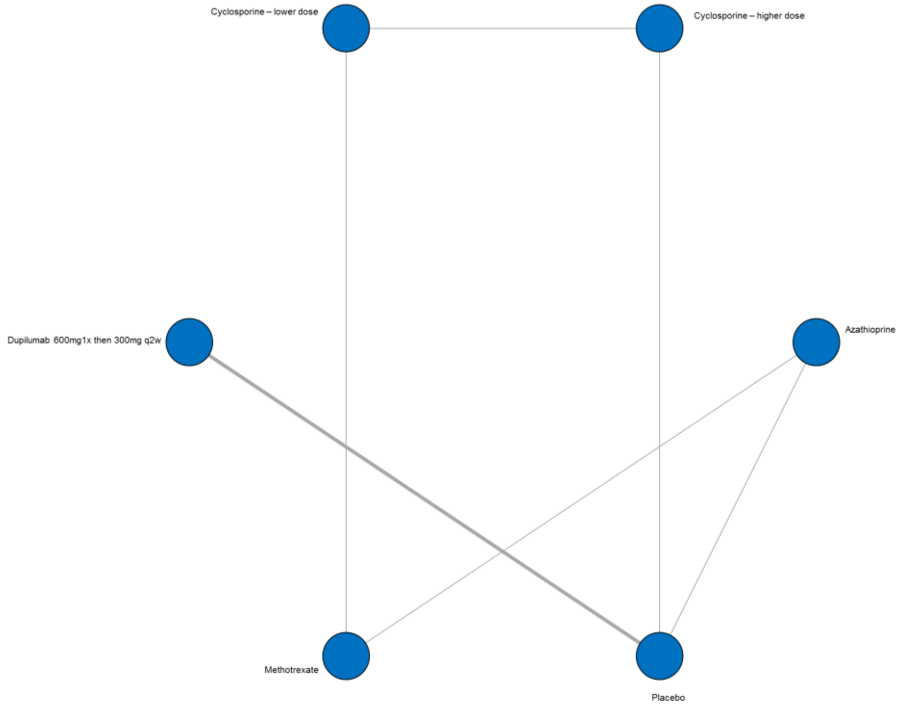

© 2020 Drucker AM et al. *JAMA Dermatology*.

eTable 2. League Table of Arms Included in the Network Meta-analysis of Change in QoL on the Standardized Mean Difference Scale up to 16 Weeks of Treatment Among Medications Currently in Use

**Azathioprine**	-0.5 (-1.3, 0.3)	-0.2 (-1, 0.5)	-0.7 (-1.3, 0)	-0.1 (-0.7, 0.6)	0.1 (-0.5, 0.8)
0.5 (-0.3, 1.3)	**Cyclosporine - higher dose**	0.3 (-0.3, 0.8)	-0.1 (-0.9, 0.6)	0.5 (-0.3, 1.2)	0.7 (0, 1.4)
0.2 (-0.5, 1)	-0.3 (-0.8, 0.3)	**Cyclosporine - lower dose**	-0.4 (-1.2, 0.4)	0.2 (-0.4, 0.7)	0.4 (-0.4, 1.1)
0.7 (0, 1.3)	0.1 (-0.6, 0.9)	0.4 (-0.4, 1.2)	**Dupilumab 600mg 1x then 300mg q2w**	0.6 (-0.2, 1.4)	0.8 (0.6, 1)
0.1 (-0.6, 0.7)	-0.5 (-1.2, 0.3)	-0.2 (-0.7, 0.4)	-0.6 (-1.4, 0.2)	**Methotrexate**	0.2 (-0.5, 1)
-0.1 (-0.8, 0.5)	-0.7 (-1.4, 0)	-0.4 (-1.1, 0.4)	-0.8 (-1, -0.6)	-0.2 (-1, 0.5)	**Placebo**

© 2020 Drucker AM et al. *JAMA Dermatology*.

eFigure 4. Network Plot of Arms Included in the Network Meta-analysis of Change in Itch on the Standardized Mean Difference Scale up to 16 Weeks of Treatment – Among Medications Currently in Use

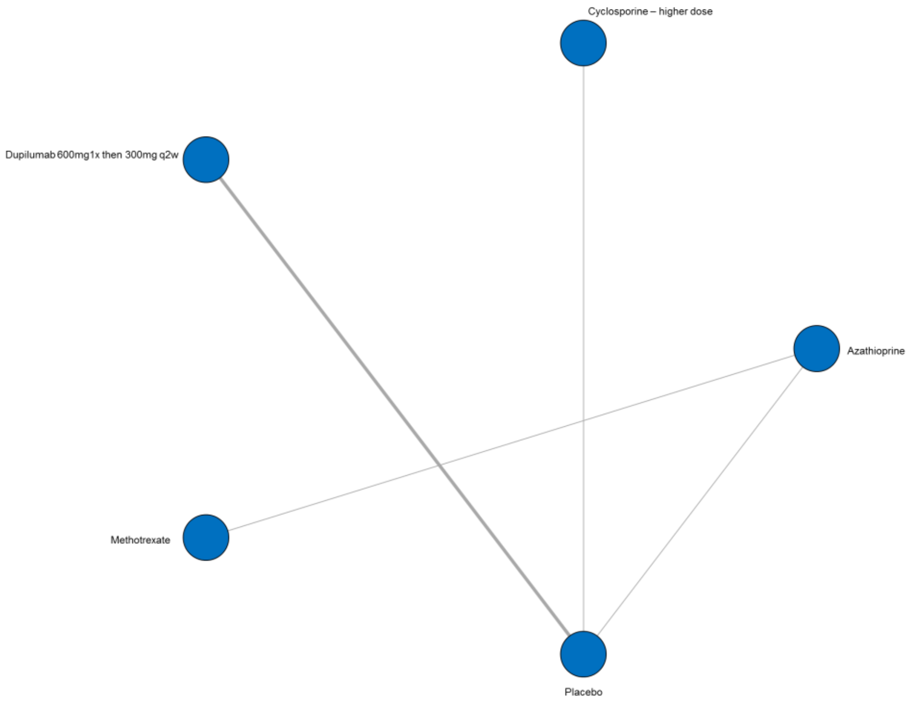

© 2020 Drucker AM et al. *JAMA Dermatology*.

eTable 3. League Table of Arms Included in the Network Meta-analysis of Change in Itch on the Standardized Mean Difference Scale up to 16 Weeks of Treatment Among Medications Currently in Use

Results are presented as the mean difference (95% CrI) in itch between the two arms on the standardized mean difference. A positive effect estimate in a given cell favors the row-defining treatment. A negative effect estimate in a given cell favors the column-defining treatment.

**Azathioprine**	-0.5 (-1.9, 0.8)	-0.6 (-1.5, 0.3)	0 (-0.8, 0.9)	0.3 (-0.6, 1.1)
0.5 (-0.8, 1.9)	**Cyclosporine - higher dose**	0 (-1.2, 1)	0.6 (-1.1, 2.2)	0.8 (-0.3, 1.8)
0.6 (-0.3, 1.5)	0 (-1, 1.2)	**Dupilumab 600mg 1x then 300mg q2w**	0.6 (-0.6, 1.9)	0.8 (0.5, 1.2)
0 (-0.9, 0.8)	-0.6 (-2.2, 1.1)	-0.6 (-1.9, 0.6)	**Methotrexate**	0.2 (-1, 1.4)
-0.3 (-1.1, 0.6)	-0.8 (-1.8, 0.3)	-0.8 (-1.2, -0.5)	-0.2 (-1.4, 1)	**Placebo**

© 2020 Drucker AM et al. *JAMA Dermatology*.

eReferences

1. Sowden JM, Berth-Jones J, Ross JS, et al. Double-blind, controlled, crossover study of cyclosporine in adults with severe refractory atopic dermatitis. Lancet 1991; 338(8760): 137-40.
2. Hanifin JM, Schneider LC, Leung DY, et al. Recombinant interferon gamma therapy for atopic dermatitis. Journal of the American Academy of Dermatology 1993; 28(2 Pt 1): 189-97.
3. Munro CS, Levell NJ, Shuster S, Friedmann PS. Maintenance treatment with cyclosporine in atopic eczema. The British journal of dermatology 1994; 130(3): 376-80.
4. Czech W, Brautigam M, Weidinger G, Schopf E. A body-weight-independent dosing regimen of cyclosporine microemulsion is effective in severe atopic dermatitis and improves the quality of life. Journal of the American Academy of Dermatology 2000; 42(4): 653-9.
5. Jang IG, Yang JK, Lee HJ, et al. Clinical improvement and immunohistochemical findings in severe atopic dermatitis treated with interferon gamma. Journal of the American Academy of Dermatology 2000; 42(6): 1033-40.
6. Granlund H, Erkko P, Remitz A, et al. Comparison of cyclosporine and UVAB phototherapy for intermittent one-year treatment of atopic dermatitis. Acta Derm Venereol 2001; 81(1): 22-7.
7. Berth-Jones J, Takwale A, Tan E, et al. Azathioprine in severe adult atopic dermatitis: a double-blind, placebo-controlled, crossover trial. The British journal of dermatology 2002; 147(2): 324-30.
8. Pacor ML, Di Lorenzo G, Martinelli N, Mansueto P, Rini GB, Corrocher R. Comparing tacrolimus ointment and oral cyclosporine in adult patients affected by atopic dermatitis: a randomized study. Clin Exp Allergy 2004; 34(4): 639-45.
9. Bemanian MH, Movahedi M, Farhoudi A, et al. High doses intravenous immunoglobulin versus oral cyclosporine in the treatment of severe atopic dermatitis. Iranian journal of allergy, asthma, and immunology 2005; 4(3): 139-43.
10. Meggitt SJ, Gray JC, Reynolds NJ. Azathioprine dosed by thiopurine methyltransferase activity for moderate-to-severe atopic eczema: a double-blind, randomised controlled trial. Lancet 2006; 367(9513): 839-46.
11. Jee SJ, Kim JH, Baek HS, Lee HB, Oh JW. Long-term Efficacy of Intravenous Immunoglobulin Therapy for Moderate to Severe Childhood Atopic Dermatitis. Allergy Asthma Immunol Res 2011; 3(2): 89-95.
12. Schram ME, Roekevisch E, Leeflang MM, Bos JD, Schmitt J, Spuls PI. A randomized trial of methotrexate versus azathioprine for severe atopic eczema. The Journal of allergy and clinical immunology 2011; 128(2): 353-9.
13. El-Khalawany MA, Hassan H, Shaaban D, Ghonaim N, Eassa B. Methotrexate versus cyclosporine in the treatment of severe atopic dermatitis in children: a multicenter experience from Egypt. Eur J Pediatr 2013; 172(3): 351-6.
14. Iyengar SR, Hoyte EG, Loza A, et al. Immunologic effects of omalizumab in children with severe refractory atopic dermatitis: a randomized, placebo-controlled clinical trial. International archives of allergy and immunology 2013; 162(1): 89-93.
15. Beck LA, Thaci D, Hamilton JD, et al. Dupilumab treatment in adults with moderate-to-severe atopic dermatitis. The New England journal of medicine 2014; 371(2): 130-9.
16. Tsianakas A, Luger TA, Radin A. Dupilumab treatment improves quality of life in adult patients with moderate-to-severe atopic dermatitis: results from a randomized, placebo-controlled clinical trial. The British journal of dermatology 2018; 178(2): 406-14.
17. Khattri S, Brunner PM, Garcet S, et al. Efficacy and safety of ustekinumab treatment in adults with moderate-to-severe atopic dermatitis. Exp Dermatol 2016.
18. Simpson EL, Bieber T, Guttman-Yassky E, et al. Two Phase 3 Trials of Dupilumab versus Placebo in Atopic Dermatitis. The New England journal of medicine 2016; 375(24): 2335-48.
19. Thaci D, Simpson EL, Beck LA, et al. Efficacy and safety of dupilumab in adults with moderate-to-severe atopic dermatitis inadequately controlled by topical treatments: a randomised, placebo-controlled, dose-ranging phase 2b trial. Lancet 2016; 387(10013): 40-52.
20. Simpson EL, Gadkari A, Worm M, et al. Dupilumab therapy provides clinically meaningful improvement in patient-reported outcomes (PROs): A phase IIb, randomized, placebo-controlled, clinical trial in adult patients with moderate to severe atopic dermatitis (AD). Journal of the American Academy of Dermatology 2016; 75(3): 506-15.

© 2020 Drucker AM et al. *JAMA Dermatology*.

21. Blauvelt A, de Bruin-Weller M, Gooderham M, et al. Long-term management of moderate-to-severe atopic dermatitis with dupilumab and concomitant topical corticosteroids (LIBERTY AD CHRONOS): a 1-year, randomised, double-blinded, placebo-controlled, phase 3 trial. Lancet 2017; 389(10086): 2287-303.
22. Goujon C, Viguier M, Staumont-Salle D, et al. Methotrexate Versus Cyclosporine in Adults with Moderate-to-Severe Atopic Dermatitis: A Phase III Randomized Noninferiority Trial. J Allergy Clin Immunol Pract 2017.
23. Ruzicka T, Hanifin JM, Furue M, et al. Anti-Interleukin-31 Receptor A Antibody for Atopic Dermatitis. The New England journal of medicine 2017; 376(9): 826-35.
24. Saeki H, Kabashima K, Tokura Y, et al. Efficacy and safety of ustekinumab in Japanese patients with severe atopic dermatitis: a randomized, double-blind, placebo-controlled, phase II study. The British journal of dermatology 2017; 177(2): 419-27.
25. De Bruin-Weller M, Guttman-Yassky E, Forman S, et al. Effects of upadacitinib on atopic dermatitis signs, symptoms and patient-reported outcomes from a phase IIb randomized, placebo-controlled trial. BRITISH JOURNAL OF DERMATOLOGY; 2018: WILEY 111 RIVER ST, HOBOKEN 07030-5774, NJ USA; 2018. p. E13-E.
26. Reich K, Guttman-Yassky E, Beck L, Hu X, Pangan A, Teixeira H. Early response to upadacitinib in moderate-to-severe atopic dermatitis: Results from a phase 2b randomized, placebo-controlled trial. Allergy; 2018: WILEY 111 RIVER ST, HOBOKEN 07030-5774, NJ USA; 2018. p. 76-.
27. de Bruin-Weller M, Thaci D, Smith CH, et al. Dupilumab with concomitant topical corticosteroid treatment in adults with atopic dermatitis with an inadequate response or intolerance to ciclosporin A or when this treatment is medically inadvisable: a placebo-controlled, randomized phase III clinical trial (LIBERTY AD CAFE). The British journal of dermatology 2018; 178(5): 1083-101.
28. Guttman-Yassky E, Brunner PM, Neumann AU, et al. Efficacy and safety of fezakinumab (an IL-22 monoclonal antibody) in adults with moderate-to-severe atopic dermatitis inadequately controlled by conventional treatments: A randomized, double-blind, phase 2a trial. Journal of the American Academy of Dermatology 2018; 78(5): 872-81 e6.
29. Guttman-Yassky E, Silverberg JI, Nemoto O, et al. Baricitinib in adult patients with moderate-to-severe atopic dermatitis: a phase 2 parallel, double-blinded, randomized placebo-controlled multiple-dose study. Journal of the American Academy of Dermatology 2018; 80(4): 913-21.e9.
30. Simpson EL, Flohr C, Eichenfield LF, et al. Efficacy and safety of lebrikizumab (an anti-IL-13 monoclonal antibody) in adults with moderate-to-severe atopic dermatitis inadequately controlled by topical corticosteroids: A randomized, placebo-controlled phase II trial (TREBLE). Journal of the American Academy of Dermatology 2018; 78(5): 863-71 e11.
31. Wollenberg A, Howell MD, Guttman-Yassky E, et al. Treatment of atopic dermatitis with tralokinumab, an anti-IL-13 mAb. The Journal of allergy and clinical immunology 2019; 143(1): 135-41.
32. Blauvelt A, Simpson EL, Tyring SK, et al. Dupilumab does not affect correlates of vaccine-induced immunity: A randomized, placebo-controlled trial in adults with moderate-to-severe atopic dermatitis. Journal of the American Academy of Dermatology 2019; 80(1): 158-67 e1.
33. Guttman-Yassky E, Bissonnette R, Ungar B, et al. Dupilumab progressively improves systemic and cutaneous abnormalities in patients with atopic dermatitis. Journal of Allergy and Clinical Immunology 2019; 143(1): 155-72.
34. Guttman-Yassky E, Pavel AB, Zhou L, et al. GBR 830, an anti-OX40, improves skin gene signatures and clinical scores in patients with atopic dermatitis. The Journal of allergy and clinical immunology 2019; 144(2): 482-93.e7.
35. Simpson EL, Imafuku S, Poulin Y, et al. A Phase 2 Randomized Trial of Apremilast in Patients with Atopic Dermatitis. The Journal of investigative dermatology 2019; 139(5): 1063-72.
36. Simpson EL, Parnes JR, She D, et al. Tezepelumab, an anti-thymic stromal lymphopoietin monoclonal antibody, in the treatment of moderate to severe atopic dermatitis: A randomized phase 2a clinical trial. Journal of the American Academy of Dermatology 2019; 80(4): 1013-21.
37. Werfel T, Layton G, Yeadon M, et al. Efficacy and safety of the histamine H4 receptor antagonist ZPL-3893787 in patients with atopic dermatitis. The Journal of allergy and clinical immunology 2019; 143(5): 1830-7 e4.
38. Efficacy and Safety Study of QAW039 in the Treatment of Patients With Moderate to Severe Atopic Dermatitis. https://ClinicalTrials.gov/show/NCT01785602.
39. Study To Evaluate Pf-04965842 In Subjects With Moderate To Severe Atopic Dermatitis. https://ClinicalTrials.gov/show/NCT02780167.

© 2020 Drucker AM et al. *JAMA Dermatology*.

40. Gooderham MJ, Forman SB, Bissonnette R, et al. Efficacy and Safety of Oral Janus Kinase 1 Inhibitor Abrocitinib for Patients With Atopic Dermatitis: A Phase 2 Randomized Clinical Trial. JAMA dermatology 2019; [Epub ahead of print].
41. Efficacy and Safety of Dupilumab in Participants ≥12 to <18 Years of Age, With Moderate-to-severe Atopic Dermatitis. https://ClinicalTrials.gov/show/NCT03054428.
42. Paller A, Blauvelt A, Pariser D, et al. Dupilumab for adolescents with moderate-to-severe atopic dermatitis: Results from a phase 3, randomized, double-blinded trial: 10051. Journal of the American Academy of Dermatology 2019; 81(4).
43. Dose-ranging Study of Nemolizumab in Atopic Dermatitis. https://ClinicalTrials.gov/show/NCT03100344.
44. Silverberg JI, Pinter A, Pulka G, et al. Phase 2B randomized study of nemolizumab in adults with moderate-to-severe atopic dermatitis and severe pruritus. The Journal of allergy and clinical immunology 2019; [Epub ahead of print].

© 2020 Drucker AM et al. *JAMA Dermatology*.
